# Machine learning potential predictor of idiopathic pulmonary fibrosis

**DOI:** 10.3389/fgene.2024.1464471

**Published:** 2025-01-22

**Authors:** Chenchun Ding, Quan Liao, Renjie Zuo, Shichao Zhang, Zhenzhen Guo, Junjie He, Ziwei Ye, Weibin Chen, Sunkui Ke

**Affiliations:** ^1^ Department of Thoracic Surgery, Zhongshan Hospital of Xiamen University, School of Medicine, Xiamen University, Xiamen, Fujian, China; ^2^ Department of Urology, Tianjin Institute of Urology, The Second Hospital of Tianjin Medical University, Tianjin, China; ^3^ School of Pharmaceutical Sciences, Xiamen University, Xiamen, Fujian, China

**Keywords:** bioinformatics, biomarkers, immune cell infiltration, machine-learning, idiopathic pulmonary fibrosis

## Abstract

**Introduction:**

Idiopathic pulmonary fibrosis (IPF) is a severe chronic respiratory disease characterized by treatment challenges and poor prognosis. Identifying relevant biomarkers for effective early-stage risk prediction is therefore of critical importance.

**Methods:**

In this study, we obtained gene expression profiles and corresponding clinical data of IPF patients from the GEO database. GO enrichment and KEGG pathway analyses were performed using R software. To construct an IPF risk prediction model, we employed LASSO-Cox regression analysis and the SVM-RFE algorithm. PODNL1 and PIGA were identified as potential biomarkers associated with IPF onset, and their predictive accuracy was confirmed using ROC curve analysis in the test set. Furthermore, GSEA revealed enrichment in multiple pathways, while immune function analysis demonstrated a significant correlation between IPF onset and immune cell infiltration. Finally, the roles of PODNL1 and PIGA as biomarkers were validated through *in vivo* and *in vitro* experiments using qRT-PCR, Western blotting, and immunohistochemistry.

**Results:**

These findings suggest that PODNL1 and PIGA may serve as critical biomarkers for IPF onset and contribute to its pathogenesis.

**Discussion:**

This study highlights their potential for early biomarker discovery and risk prediction in IPF, offering insights into disease mechanisms and diagnostic strategies.

## 1 Introduction

Recent investigations have reported that the global incidence of idiopathic pulmonary fibrosis (IPF) ranges from 1 to 13 cases per 100,000 individuals, with a prevalence of 3–45 cases per 100,000 individuals (Anna et al., 2023). The median survival time for patients with IPF is approximately 3–5 years. Early diagnosis primarily relies on imaging assessments; however, 20%–25% of patients exhibit atypical imaging features, underscoring the limitations of current clinical diagnostic methods. According to the clinical practice guidelines issued by the American Thoracic Society/European Respiratory Society/Japanese Respiratory Society/Asociación Latinoamericana de Tórax (ATS/ERS/JRS/ALAT), no serum biomarkers are currently recommended for monitoring IPF progression (Weiwei et al., 2024). Recent clinical studies have demonstrated that the Envisia Genomic Classifier (EGC) is an effective molecular diagnostic tool for identifying usual interstitial pneumonia (UIP) patterns via bronchoscopy, aiding in the accurate diagnosis and management of IPF (Lisa et al., 2022). Additionally, several genes, including IL18R1, m5CPS, and CYFRA 21–1, have been identified as potential biomarkers for the diagnosis, prognosis, and treatment of IPF (Philip et al., 2022; Tao and Hua-Fu, 2022; Kun et al., 2023). The discovery and validation of new biomarkers are crucial for accurately predicting disease outcomes, assessing disease severity, and identifying patients with poor prognoses at early stages, which will significantly benefit clinical practice.

Biomarker screening typically involves analyzing large-scale datasets, including gene expression, protein, and metabolite data. The high dimensionality of these datasets often poses challenges for traditional statistical methods, which may struggle to handle complex and nonlinear relationships between disease occurrence and progression. In contrast, machine learning algorithms can effectively identify such nonlinear patterns, offering a powerful tool for discovering novel and effective disease biomarkers. Machine learning, an automated data analysis method for constructing predictive models, has seen widespread application in clinical medicine (Wenxin et al., 2021; Reena et al., 2024). Previous studies have shown that machine learning can predict the risk of diseases such as breast and endometrial cancer, identify histopathological features, and enrich related biological pathways (Stephen-John et al., 2021; Woong–Chul and Sen, 2023).

In recent years, machine learning has also been applied extensively to the diagnosis and treatment of various diseases (Iain et al., 2023). For example, the Least Absolute Shrinkage and Selection Operator (LASSO) logistic regression combines multiple decision trees iteratively constructed from random subsets of predictor and outcome variables to enhance predictive accuracy (Shihu et al., 2021). Similarly, support vector machine recursive feature elimination (SVM-RFE) is widely used to select optimal variable combinations, leveraging its nonlinear discriminative characteristics (Min et al., 2023). Using machine learning, Wu et al. identified FHL2, HPCAL1, RNF182, and SLAIN1 as potential biomarkers for IPF (Zenan et al., 2023). Given the complexity of IPF as a multifaceted disease, discovering and validating additional biomarkers will enhance our understanding of its underlying mechanisms and improve diagnostic accuracy. In summary, machine learning not only improves the efficiency and accuracy of biomarker discovery but also offers novel insights for the diagnosis, treatment, and prevention of diseases. The application of machine learning to identify IPF biomarkers is, therefore, of significant importance.

The study of the immune cell landscape in IPF holds substantial scientific and clinical relevance (Eddy et al., 2024). The immune system plays a pivotal role in the initiation and progression of fibrosis, with immune cells such as macrophages, T cells, B cells, and dendritic cells closely associated with the pathological changes observed in IPF (Kevin et al., 2021; Cecilia et al., 2022). The interactions, migration, and responses of immune cell subsets to cytokines and growth factors may be central to the immune dysregulation and fibrotic processes involved in IPF (Yahan et al., 2023). Recent studies have further demonstrated the complex behaviors of immune cells in the tumor microenvironment, particularly in malignancies such as hepatocellular carcinoma (HCC) and glioblastoma (GBM), where immune dynamics significantly impact disease prognosis and treatment outcomes (Guimei et al., 2020; Tianqi et al., 2021). Thus, in-depth exploration of the immune cell landscape may not only enhance our understanding of the immune mechanisms underlying IPF but also identify novel biomarkers, thereby improving early diagnostic capabilities and disease progression predictions.

In this study, we developed risk prediction models based on the key genes PODNL1 and PIGA. Gene Set Enrichment Analysis (GSEA) was employed to investigate the biological characteristics and molecular pathways associated with IPF. Additionally, immune cell profiling was performed to examine the relationship between hub genes and the immune landscape in disease contexts. These findings were validated through *in vivo* and *in vitro* experiments, offering further evidence supporting the reliability of the IPF risk prediction model and advancing our understanding of the molecular and immune mechanisms underlying IPF.

## 2 Materials and methods

### 2.1 Data acquisition

Data were from the GEO dataset (GEO, https://www.ncbi.nlm.nih.gov/geo/database). The GSE21369 dataset, consisting of 23 IPF samples and 6 normal samples (Ji-Hoon et al., 2011), and the GSE10667 dataset, consisting of 31 IPF samples and 15 normal samples (Iván et al., 2008), were utilized in this study. Differentially Expressed Genes (DEGs) were identified using the aforementioned GEO dataset.

### 2.2 Differentially expressed genes (DEGs) analysis

We used R 4.4.1 software for DEGs screening, data processing, and DEG analysis, employing the “DESeq2” package in R software (Zitao et al., 2022). Visualization of DEGs was conducted using the “pheatmap” and “ggplot2” packages, producing volcano plots and heatmaps, respectively (Jiannan et al., 2024).

### 2.3 Functional enrichment analysis

To explore the potential mechanisms of DEGs in IPF, the “clusterProfiler” package was utilized for Gene Ontology (GO), Disease Ontology (DO) and Kyoto Encyclopedia of Genes and Genomes (KEGG) enrichment analysis (Wenyuan et al., 2023; Dongliang et al., 2024). Statistical significance was defined as P_FDR_ values less than 0.05 for both KEGG and GO enrichment analyses.

### 2.4 Protein‒protein interaction (PPI) network construction

DEGs and other genes were annotated with the help of the Search Tool for the Retrieval of Interacting Genes (STRING) online database (http://string-db.org) (Limin et al., 2022). The PPI network was constructed using only those interactions that had been empirically validated and had a total score that was higher than 0.4 (Changjin et al., 2024).

### 2.5 Candidate biomarker screening

This study employed two machine learning algorithms to identify feature genes associated with IPF, including LASSO logistic regression and SVM-RFE (Yizhong et al., 2019). LASSO logistic regression analysis was performed using the “glmnet” package in R software (Miduo et al., 2022); SVM-RFE algorithm was implemented using the “e107”package in R software. Feature genes identified by LASSO logistic regression and SVM-RFE algorithms intersected to generate potential biomarkers. Furthermore, the accuracy of biomarkers was evaluated through ROC curve analysis on training and testing datasets using the “pROC” package (Qiyu et al., 2023). GSE53845 and GSE10667 are used as external data for the training and testing datasets, respectively (Iván et al., 2008; Daryle et al., 2014).

### 2.6 Cell culture

A549 cells were purchased from the ATCC and maintained in DMEM high-glucose medium (C7076-500mL, Bioss) supplemented with 10% fetal bovine serum and 1% penicillin/streptomycin. Cells were cultured at 37°C in a 5% CO_2_ atmosphere. Cells were seeded at a density of 2.5 × 10^4^ cells and passaged regularly. Boermycin (11-B608166, Boer).

### 2.7 Quantitative real-time PCR analysis

Total RNA was extracted from A549 cells and lung tissues using TRlzol reagent (YZ-15596018, Acmec). The concentration of total RNA was measured using a NanoDrop One ultramicro spectrophotometer (Thermo). cDNA was synthesized using Hifair^®^ II Reverse Transcriptase (11110ES92*, Yeasen), and qPCR was performed using Hieff^®^ qPCR SYBR Green Master Mix (11203ES08, Yeasen). β-Actin was used as an internal control, and data were normalized to the control. Baijin Biotechnology provided the primers that were used in this study. The normalizer employed in this study was GAPDH. The following primers were used for qPCR: Human GAPDH: 5′-GGA​GCG​AGA​TCC​CTC​CAA​AAT-3′ and 5′-GGCTGT TGTCAT ACT TCT CAT GG-3′; Human PODNL1: 5′-AGA​CAT​CAT​CCC​CCA​GCT​CT-3′ and 5′-GCT​CGG​CCA​CTG​GGT​G-3′; Human PIGA: 5′- GCC​ATG​GAA​CTC​ACC​GGT​AAT​AGA -3′ and 5′- AGA​GTG​TAG​CTG​AGG​CAC​GG -3′; Human Sftpc: 5′- GCT​ACA​GCC​TAA​GGG​CAA​CA -3′ and 5′- GGG​ATC​ACA​CCT​GCT​CAC​C -3′; Human Sftpa1: 5′- ACT​TGG​AGG​CAG​AGA​CCC​AA -3′ and 5′- GGC​TTC​CAA​CAC​AAA​CGT​CC -3′; Human Collagen1a1: 5′- CGA​GGC​TCT​GAA​GGT​CCC​C -3′ and 5′- CCAGGAGCACCATTGGCA -3′; Human Fibronectin: 5′- AAG​AAG​GGC​TCG​TGT​GAC​AG -3′ and 5′- TCT​TGT​CCT​ACA​TTC​GGC​GG -3′.

### 2.8 Western blot analysis

Total protein was extracted using RIPA lysis buffer with protease and phosphatase inhibitors. Protein concentrations were determined using the BCA assay (BX-2142728, Pierce). Proteins were separated by SDS-PAGE and transferred to PVDF membranes. The membranes were blocked with 5% skim milk, incubated with antibodies overnight at 4°C, washed, and incubated with secondary antibodies. Band intensities were detected using a chemiluminescence imager (Biorad). The primary antibodies used included PODNL1 (1:500, AP12207c, ABGENT), PIGA (1:2000, ab69768, Abcam), Sftpc (1:1000, ab312851, Abcam), Sftpa1 (1:1000, ab190087, Abcam),Collagen1a1 (1:1000, ab138492, Abcam) and Fibronectin (1:1000, ab2413, Abcam).

### 2.9 Plasmid and cell transfection

The overexpression plasmid vector was designed and provided by GenePharma Technologies (China). When the cultured cell density reached 70%, the cells were washed with serum-free medium and then serum-free medium was added, followed by the addition of a transfection reagent. After 24 h of incubation, the transfection solution was poured out and replaced with complete medium for continued cultivation. Three days later, mRNA and protein levels were measured, and subsequent experiments were conducted.

### 2.10 Animals

This study utilized male C57BL/6 mice (6 weeks old) obtained from the Experimental Animal Center of Xiamen University. The animal study was reviewed and approved by Animal Ethics Committee of Xiamen University (Ethical code: XMULAC20240136). Mice were anesthetized with 1% pentobarbital sodium (60 mg/kg), followed by intratracheal administration of 2 mg/kg bleomycin (11-B608166, Boer) dissolved in 40 μL of sterile saline to induce pulmonary fibrosis model. Subsequently, mice were euthanized on the 20th day post-bleomycin administration, and lung tissue specimens were collected.

### 2.11 Immunohistochemistry (IHC)

Lung tissues were obtained from mouse models. Following paraffin embedding, the samples were cut into 5-µm-thick slices with a microtome and IHC was performed. The primary antibodies against PODNL1 and PIGA were diluted and incubated overnight at 4°C with the tissue sections. Following that, the tissue sections were treated with an immunohistochemical reagent (KIT-9720, MXB, China) according to the manufacturer’s instructions. After staining the slice with diaminobenzidine (DAB) solution (Servicebio, China), the slides were mounted and examined under a light microscope (Nikon SMZ 1000). The primary antibodies used included PODNL1 (1:50, AP12207c, ABGENT) and PIGA (1:50, 13679-1-AP, Proteintech).

### 2.12 Assays of immune cellular patterns in microenvironment

CIBERSORT is a deconvolution algorithm employed to estimate the infiltration of immune cells in both the IPF and control groups (Zitao et al., 2022). A P value of less than 0.05 was considered statistically significant. Group comparisons were conducted using the Wilcoxon rank sum test. To visualize the differences in immune cell infiltration, a violin plot was generated using the “ggplot2” package (Chong et al., 2023). Additionally, the “corrplot” package was utilized to create a correlation heatmap illustrating the relationships between immune infiltrating cells (Zhiwei et al., 2022). For our analysis, we used the LM22 matrix in CIBERSORT, which was selected due to its suitability for deconvoluting immune cell composition in immune-related studies.

### 2.13 Correlation analysis between biomarkers and infiltrating immune cells

The association between the biomarkers and the levels of immune infiltrating cells was analyzed using Spearman’s rank correlation in R software. The results were visualized using the “ggplot2” package. P values less than 0.05 were considered statistically significant (Zhiwei et al., 2022).

### 2.14 Statistical analysis

R software 4.4.1 was employed in this study. DEGs screening, data processing, and DEG analysis between IPF and normal samples using a threshold of P_FDR_ < 0.05 and |log2 Fold Change (FC)| > 1. In the volcano plot, DEGs with log2FC < 0 were considered downregulated, while those with log2FC > 0 were considered upregulated.

The SPSS 20.0 software (SPSS Inc., Chicago, IL, United States) was used for statistical analysis. The data are represented by the mean ± SD. Statistical analyses were applied using the Student’s t-test and one-way analysis of variance to determine statistical significance. Asterisks denote statistical significance (*P < 0.05, **P < 0.01, ***P < 0.001, ****P < 0.0001, ns indicates no significance). Before performing the *t*-test, we conducted the Shapiro-Wilk test to assess the normality of the data distribution.

## 3 Results

### 3.1 Identification of differentially expressed genes in idiopathic pulmonary fibrosis

**FIGURE 1:**
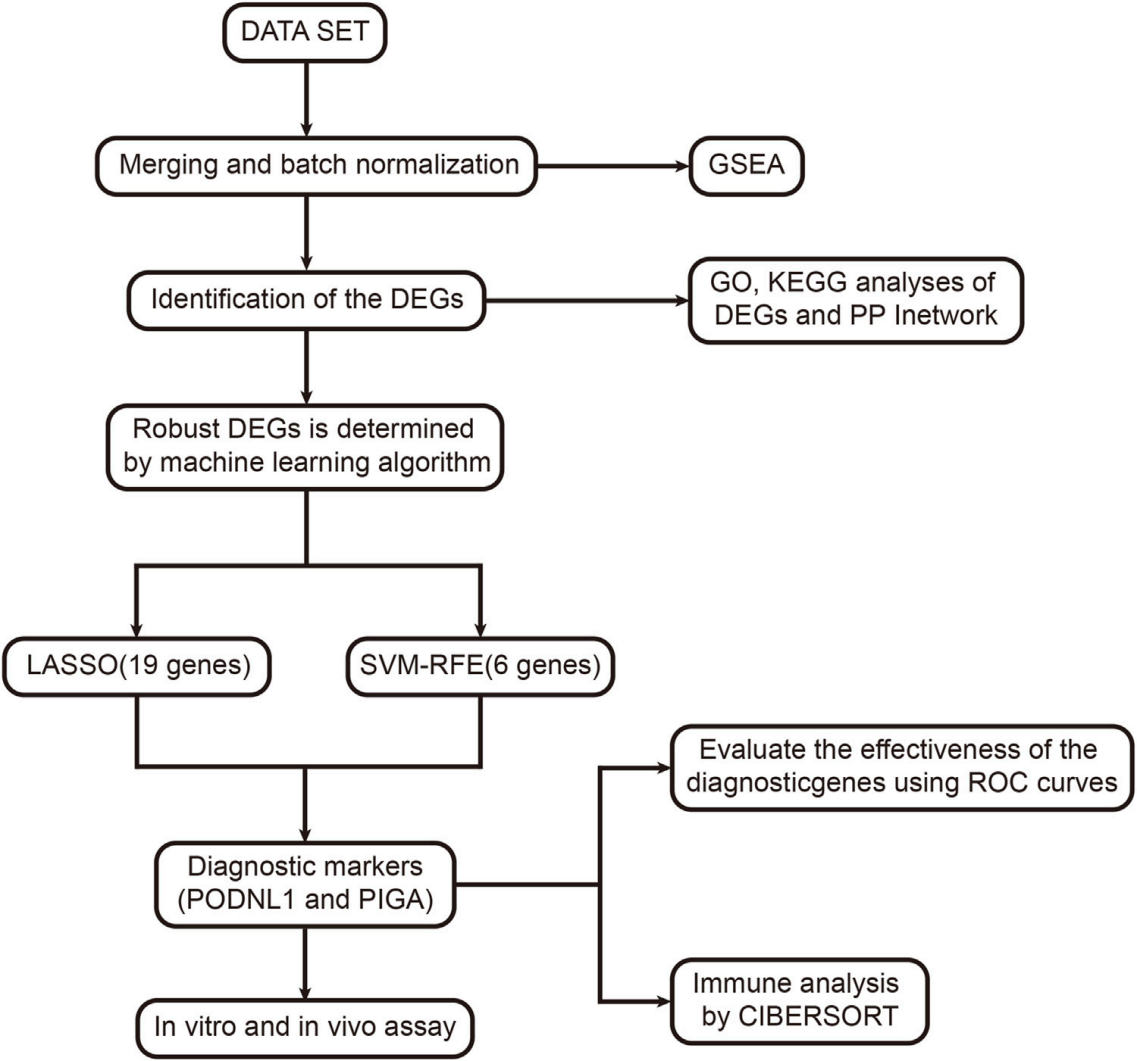
Overview of the study design.

**FIGURE 2:**
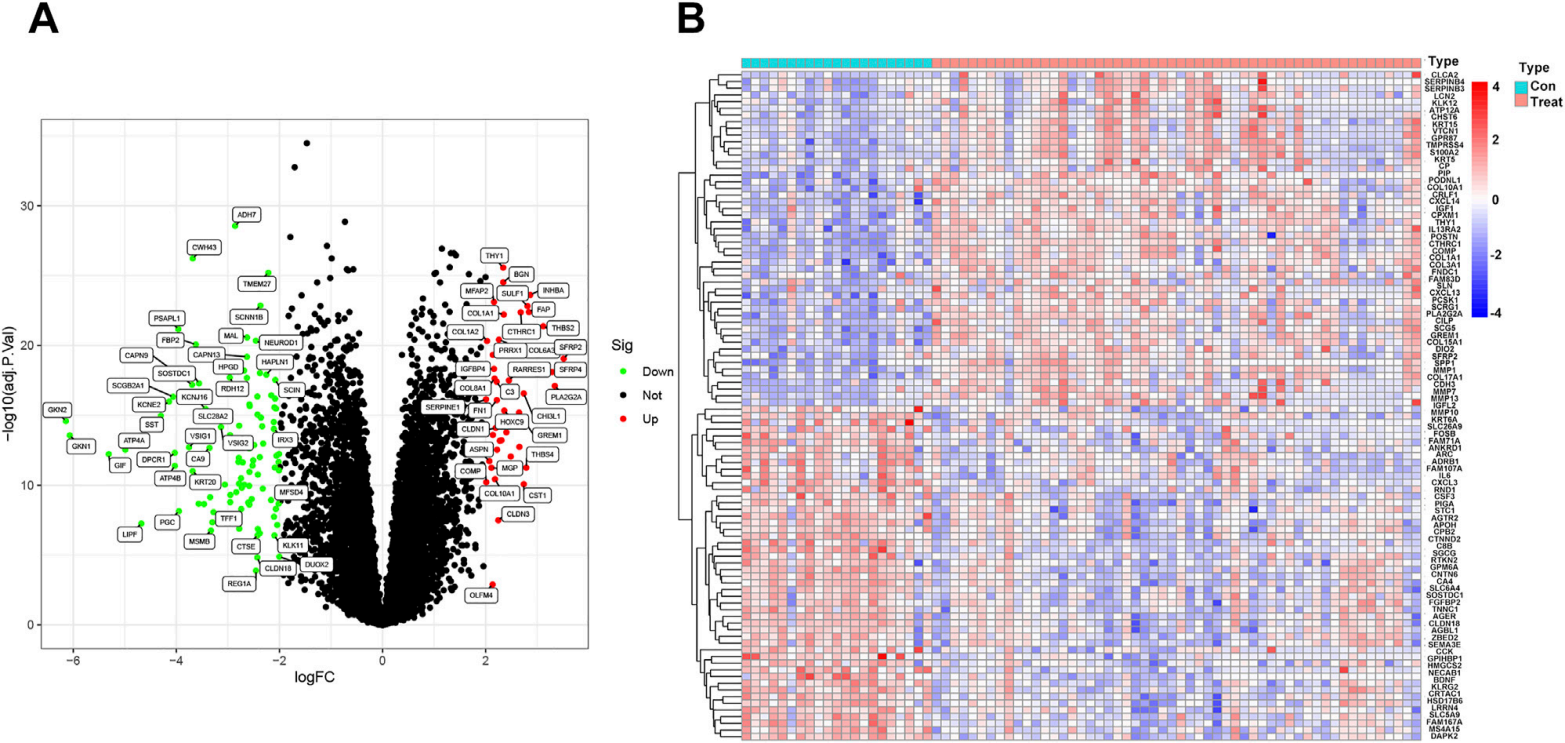
Identification of differentially expressed genes in idiopathic pulmonary fibrosis: **(A)** Volcano plot of the GSE21369 and GSE10667 dataset. **(B)** Heatmap visualization of the DEGs between idiopathic pulmonary fibrosis and normal samples. P_FDR_ < 0.05.

To visualize this study, the workflow is illustrated in (Figure 1). The differences between the two groups of samples were evaluated, identifying 2359 upregulated genes and 1299 downregulated genes (*p* < 0.05) (Figure 2A). Among them, the expression profiles of the top 50 differentially expressed genes were presented in the form of heatmap (Figure 2B).

### 3.2 Functional enrichment analysis of DEGs

**FIGURE 3:**
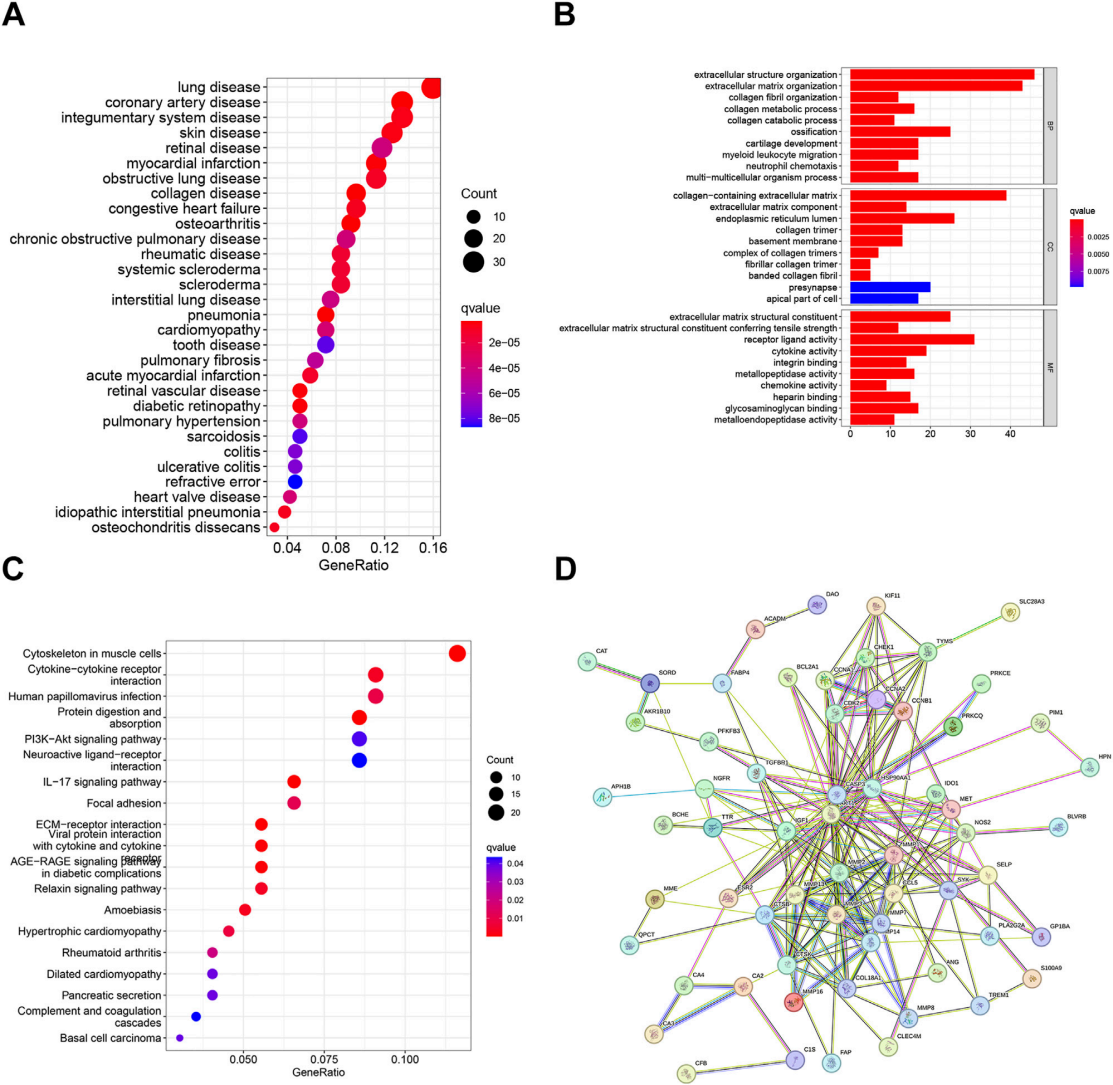
Functional enrichment analysis of DEGs: **(A)** DO analysis of DEGs. **(B)** GO analysis of DEGs. **(C)** KEGG pathway enrichment analysis of DEGs. **(D)** PPI network and hub gene identification. P_FDR_ < 0.05.

We conducted functional analysis to further investigate the biological functions of the differentially expressed genes (DEGs). The results of DO analysis revealed that these DEGs were linked to lung disease, coronary artery disease, integumentary system disease, skin disease, retinal disease, etc. (Figure 3A). The GO enrichment analysis results indicated that the DEGs were primarily enriched in biological processes such as extracellular structure organization, extracellular matrix organization, collagen fibril organization, etc. (Figure 3B). KEGG pathway enrichment analysis showed that DEGs were significantly enriched in in 19 pathways, such as cytoskeleton in muscle cells, cytokine−cytokine receptor interaction, human papillomavirus infection, Protein digestion and absorption, etc. (Figure 3C). Moreover, we analyzed DEGs using the STRING database, there were 41 nodes and 218 edges enriched in the PPI network (Figure 3D).

### 3.3 Establishment and predict value of risk model

**FIGURE 4:**
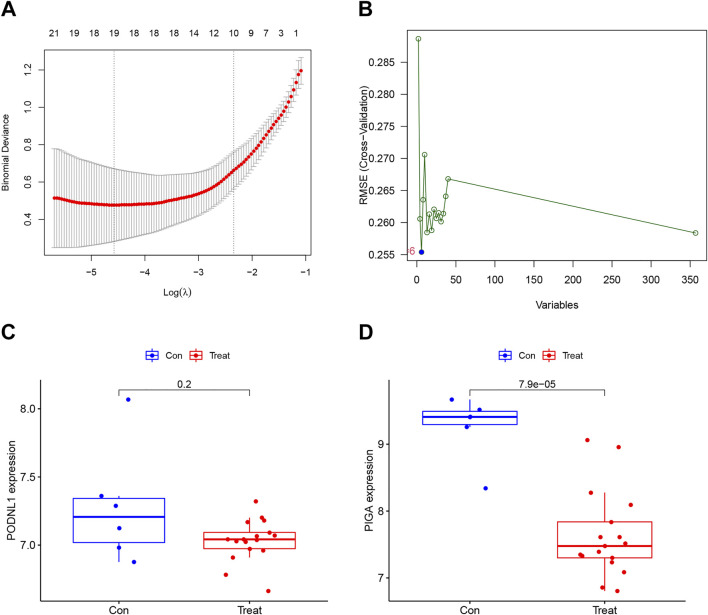
Establishment and predict value of risk model: **(A)** Characteristic genes selection via LASSO algorithm. **(B)** Characteristic genes selection via SVM-RFE algorithm. **(C, D)** Box plots of the expression of biomarkers (PODNL1 and PIGA) between normal and IPF samples in the training set (con represents normal samples, and treat represents IPF samples). P_FDR_ < 0.05.

Two validated machine learning algorithms, LASSO and SVM-RFE, were utilized to pinpoint key feature genes linked to IPF. The LASSO algorithm identified 19 feature genes (Figure 4A), while the SVM-RFE algorithm identified six feature genes as biomarkers (Figure 4B). Only the intersecting genes (PODNL1, PIGA) were ultimately selected as biomarkers for IPF. Additionally, the selected biomarkers showed good differential expression in the training sets, showing decreased expression levels of PODNL1 and PIGA in the IPF group (Figures 4C, D).

### 3.4 Diagnostic effectiveness of biomarkers

**FIGURE 5:**
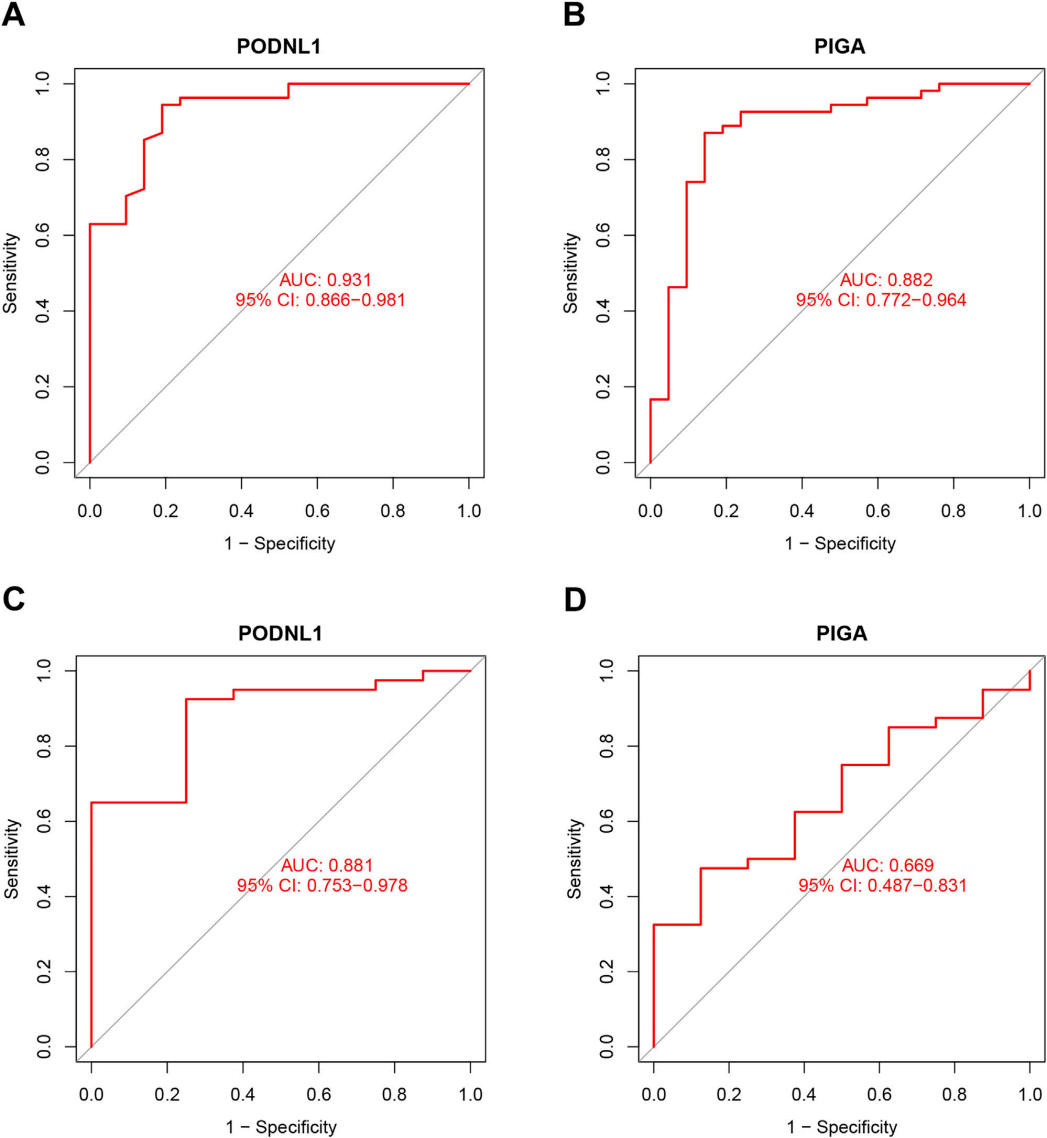
Diagnostic effectiveness of biomarkers: **(A, B)** ROC analysis was conducted for PODNL1 and PIGA in training sets. **(C, D)** ROC analysis was conducted for PODNL1 and PIGA in test sets. P_FDR_ < 0.05.

To further evaluate the diagnostic value of the identified genes in IPF, ROC analysis was performed on the four key genes in both the training and test sets. The results indicated that the four diagnostic biomarkers identified by the machine learning algorithms exhibited strong diagnostic capabilities in the training set. The AUC for PODNL1 was 0.931 (95% CI 0.866–0.981) (Figure 5A), and the AUC for PIGA was 0.882 (95% CI 0.772–0.964) (Figure 5B). Furthermore, an extra dataset (GSE53845) was used to verify the above result as a testing group. The AUC for PODNL1 was 0.881 (95% CI 0.753–0.978) (Figure 5C), and the AUC for PIGA was 0.669 (95% CI 0.487–0.831) (Figure 5D). As illustrated in the figures above, all four genes exhibited strong discriminatory ability for idiopathic pulmonary fibrosis.

### 3.5 Gene set enrichment analysis (GSEA)

**FIGURE 6:**
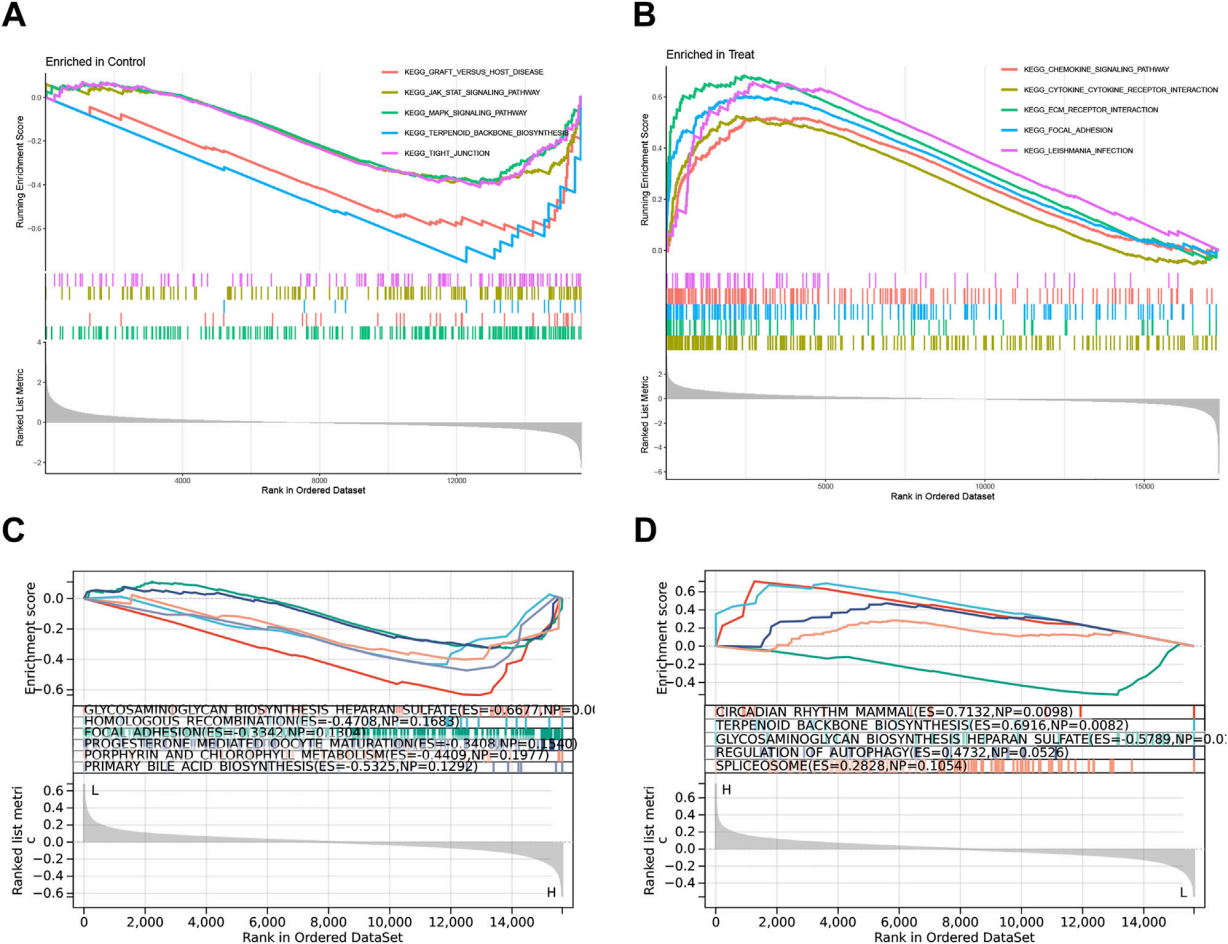
Gene Set Enrichment Analysis (GSEA): **(A, B)** The top 5 KEGG pathways enriched in the control group and IPF group. **(C, D)** The top 5 KEGG pathways enriched in the PODNL1 group and PIGA group. P_FDR_ < 0.05.

To investigate the functional characteristics of the risk model, this study performed the GO enrichment and KEGG pathway analyses between the two groups by GSEA. The top five pathways that are more prevalent in the control group include Graft versus host disease, JAK-STAT signaling pathway, MAPK signaling pathway, Terpenoid backbone biosynthesis, and Tight junction (Figure 6A), while Chemokine signaling pathway, Cytokine-cytokine receptor interaction, ECM receptor interaction, Focal adhesion, and Leishmania infection enriched in the treatment group (Figure 6B). Furthermore, to explore the roles of key genes in biological functions, we conducted KEGG pathway for PODNL1 and PIGA. In KEGG pathway analysis, PODNL1 significantly enriched pathways including Glycosaminoglycan biosynthesis heparan sulfate, Homologous recombination, Focal adhesion, Progesterone mediated oocyte maturation, Porphyrin and chlorophyll metabolism, and Primary bile acid biosynthesis (Figure 6C). PIGA enriched pathways such as Circadian rhythm - mammal, Terpenoid backbone biosynthesis, Glycosaminoglycan biosynthesis heparan sulfate, Regulation of autophagy, and Spliceosome (Figure 6D).

### 3.6 *In vivo* and vitro experiments

**FIGURE 7:**
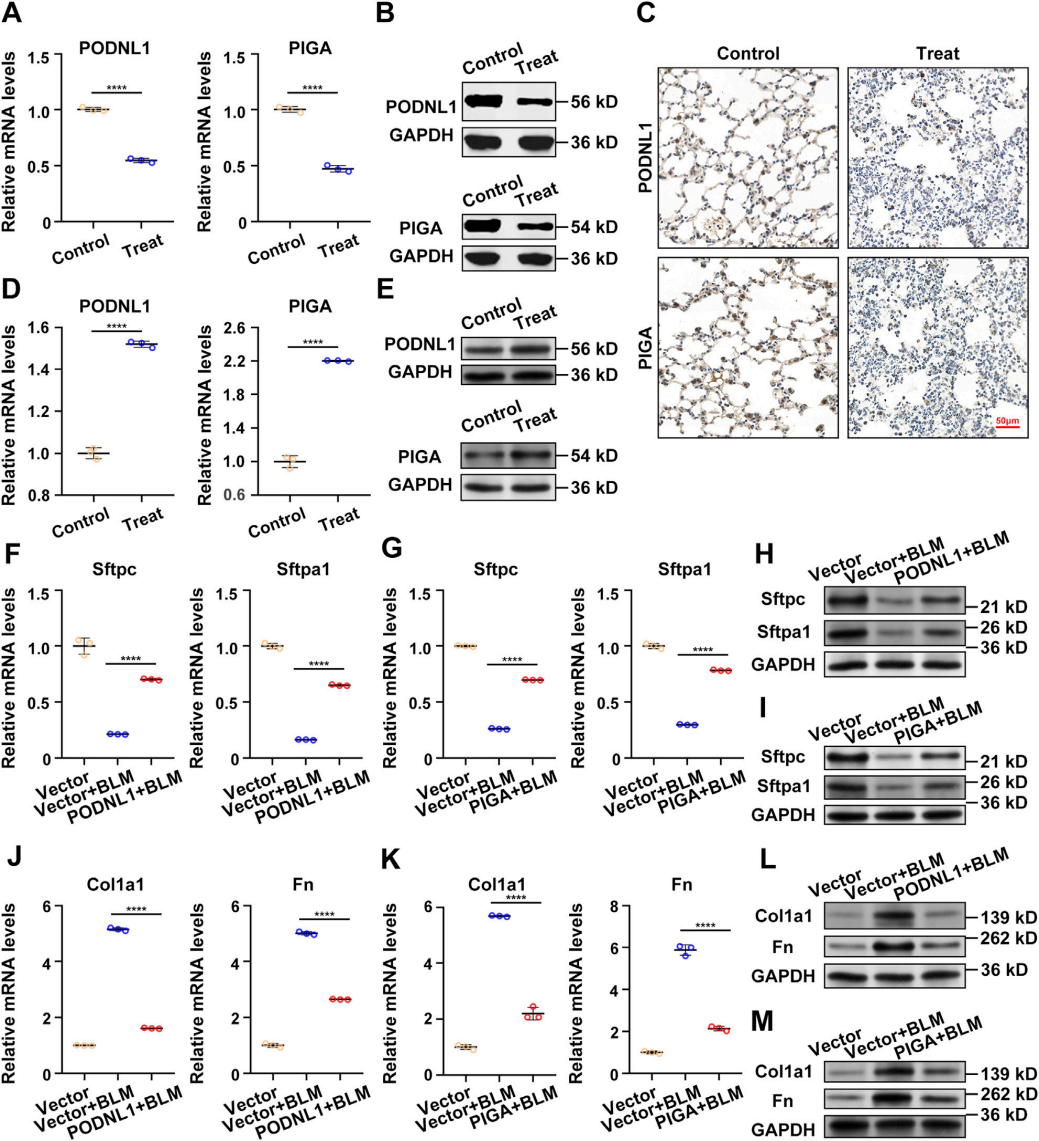
*In vivo* and vitro experiments: **(A)** RT-qPCR analysis of PODNL1 and PIGA gene expression levels in A549 cells (con represents PBS-treated group, treat represents BLM-treated group). **(B)** Western blot analysis and quantification of PODNL1 and PIGA protein levels in A549 cells. **(C)** IHC determination of PODNL1 and PIGA expression in mouse lung tissues. **(D)** The overexpression efficiency of PODNL1 and PIGA were verified by qRT–PCR in A549 cells. **(E)** The overexpression efficiency of PODNL1 and PIGA were verified by Western blot in A549 cells. **(F, G)** qRT-PCR was used to detect the effect of PODNL1 and PIGA overexpression on the mRNA levels of Sftpc and Sftpa1 in A549 cells. **(H, I)** Western blot was used to detect the effect of PODNL1 and PIGA overexpression on the expression levels of Sftpc and Sftpa1 in A549 cells. **(J, K)** qRT-PCR was used to detect the effect of PODNL1 and PIGA overexpression on the mRNA levels of Col1a1 and Fn in A549 cells. **(L, M)** Western blot was used to detect the effect of PODNL1 and PIGA overexpression on the expression levels of Col1a1 and Fn in A549 cells. Scale bars = 50 µm *P < 0.05, **P < 0.01, *** P < 0.001.

To enhance the reliability of our research findings, we developed both animal and cell models. qRT-PCR results revealed a significant decrease in the mRNA expression levels of PODNL1 and PIGA in BLM-treated A549 cells (Figure 7A). This downward trend was further confirmed by Western blot analysis, which showed reduced protein expression levels of PODNL1 and PIGA in the same cell line (Figure 7B). In the mouse model, IHC results highlighted a marked reduction in the expression of PODNL1 and PIGA in the lung tissue of IPF mice compared to the normal group (Figure 7C). Next, to verify the biological effects of PODNL1 and PIGA in A549 cells, we overexpressed the target genes respectively, and performed qRT-PCR and Western blot to assess the transfection efficiency (Figures 7D, E). Epithelial-mesenchymal transition (EMT) is a key process involved in the occurrence of IPF, characterized by insufficient regeneration of epithelial cells and increased interstitial cells. We found that overexpression of PODNL1 and PIGA can improve the reduced levels of alveolar epithelial markers Sftpc and Sftpa1 caused by BLM treatment (Figures 7F–I), while effectively inhibiting the upregulation of EMT markers Collagen1a1 (Col1a1) and Fibronectin (Fn) (Figures 7J–M). Collectively, our *in vivo* and *in vitro* experiments further substantiate the role of PODNL1 and PIGA genes in the pathogenesis of IPF, suggesting their potential as biomarkers for disease diagnosis.

### 3.7 Immune infiltration

The infiltration status of 25 types of immune cells between idiopathic pulmonary fibrosis group and control group were assessed with CIBERSORT algorithm. The percentage of the 25 types of immune cells between idiopathic pulmonary fibrosis group and control group was shown in the bar plot (Supplementary Figure S1A). The correlation of 25 types of immune cells revealed that Plasma cells was negatively related with Monocytes (r = −0.53), RMSE was negatively related with Correlation (r = −0.98), whereas RMSE was positively related to P-value (r = 0.53), P-value was positively related with T cells regulatory (Tregs) (r = 0.67) (Supplementary Figure S1B). The violin plot of the immune cell infiltration difference demonstrated that patients with idiopathic pulmonary fibrosis had a higher level of B cells memory, Plasma cells, T cells CD4 naive, Macrophages M0, Macrophages M2 and Mast cells resting compared with the control group (Supplementary Figure S1C).

### 3.8 Correlation analysis between biomarkers and immune cells

As indicated from the correlation analysis, PODNL1 was positively correlated with Correlation, Macrophages Mo, Plasma cells, B cells memory, etc., and negatively correlated with Eosinophils, etc. (Supplementary Figure S2A). PIGA was positively correlated with T cells CD4 memory resting, Eosinophils, Dendritic cells activated, Neutrophils, etc., and negatively correlated with T cells CD4 naive, etc. (Supplementary Figure S2B). PODNL1 displayed a positive correlation with Plasma cells (r = 0.28, *p* < 0.05), Monocytes (r = −0.33, *p* < 0.01), Macrophages M0 (r = 0.3, *p* < 0.01), B cells naive (r = −0.24, *p* < 0.05), Correlation (r = 0.3, *p* < 0.01), Eosinophils (r = −0.39, *p* < 0.001), B cells memory (r = 0.24, *p* < 0.05), T cells follicular helper (r = −0.24, *p* < 0.05), T cells CD4 memory resting (r = −0.25, *p* < 0.05), RMSE (r = − 0.25, *p* < 0.05), P-value (r = − 0.28, *p* < 0.05) (Supplementary Figure S3A–K). PIGA displayed a positive correlation with B cells memory (r = −0.35, *p* < 0.01), T cells CD8 (r = −0.3, *p* < 0.01), T cells CD4 naive (r = −0.38, *p* < 0.001),Plasma cells (r = −0.26, *p* < 0.05), Macrophages M2 (r = − 0.27, *p* < 0.05), NK cells resting (r = 0.23, *p* < 0.05), Neutrophils (r = 0.39, *p* < 0.001), Eosinophils (r = 0.49, *p* < 0.001), Mast cells resting (r = − 0.3, *p* = 0.01), T cells CD4 memory resting (r = 0.54, *p* < 0.001), B cells naive (r = 0.25, *p* < 0.05), Dendritic cells activated (r = 0.44, *p* < 0.001) (Supplementary Figure S3A–I). It can be concluded that PODNL1 and PIGA were correlated with immune cells.

## 4 Discussion

IPF is a progressive parenchymal lung disease that is challenging to reverse once diagnosed. The lack of sensitive diagnostic tools for early detection significantly hampers timely intervention (Richard and David, 2019). Investigating potential biomarkers involved in IPF pathogenesis could provide critical diagnostic insights during the early stages of the disease and aid in monitoring its progression. These findings will enable clinicians to identify reliable biomarkers and offer novel perspectives for future clinical research and applications in IPF diagnosis.

In this study, the GSE dataset was obtained from the GEO database to identify DEGs between IPF and normal lung tissues. GO and KEGG analyses were performed to explore the biological functions and pathways associated with these DEGs. Through a combination of LASSO logistic regression and SVM-RFE algorithms, we identified two potential biomarkers, PODNL1 and PIGA, for IPF. The diagnostic accuracy of these biomarkers was evaluated using ROC curve analysis. Furthermore, immune cell infiltration was analyzed using the CIBERSORT algorithm, revealing the relationship between infiltrating immune cells and the identified biomarkers. Expression levels of PODNL1 and PIGA were further validated in cell and mouse models, providing additional evidence for the robustness of our machine learning analysis.

Previous studies have shown that PODNL1, a member of the small leucine-rich proteoglycan family, is a potential tumor matrix-mediated biomarker and is strongly associated with glioma prognosis (Geyang et al., 2023; Shanqiang et al., 2023). Additionally, PODNL1 expression is significantly linked to the EMT pathway in bladder cancer (Xiao et al., 2022). PIGA, an enzyme involved in GPI anchor biosynthesis, has been implicated in juvenile hemochromatosis and paroxysmal nocturnal hemoglobinuria (Gregor et al., 2021). While these biomarkers have been characterized in other diseases, their high diagnostic accuracy in both the training and testing sets of our model highlights their potential utility as diagnostic targets for IPF. However, given their involvement in multiple diseases, these findings suggest potential shared pathophysiological mechanisms. Therefore, further studies are needed to elucidate their specific roles in IPF and to establish their value as disease-specific indicators.

In this study, the expression levels of PODNL1 and PIGA were validated *in vivo* and *in vitro*. Notably, their expression levels were significantly reduced in BLM-induced A549 cells and in lung tissues of BLM-induced mouse models of IPF. These results underscore the reliability of our prognostic model. Although the precise etiology of IPF remains unclear and likely multifactorial, fibrosis is consistently accompanied by innate and adaptive immune responses. Using CIBERSORT, we observed significant alterations in 25 immune cell subsets between IPF and normal tissues, further emphasizing the role of immune responses in IPF pathogenesis. In conclusion, identifying key genes involved in IPF pathogenesis not only facilitates early diagnosis and prognosis but also lays the groundwork for targeted therapeutic development. By identifying genes closely linked to disease progression, clinicians can design more effective treatment strategies and develop personalized treatment plans for IPF patients.

Despite the significant contributions of this study, some limitations remain. The molecular mechanisms underlying the identified biomarkers in IPF have not been fully elucidated and require further experimental validation. Additionally, as this study did not include clinical patient samples, the diagnostic potential of the identified biomarkers was assessed indirectly. Furthermore, the relatively weak correlation observed between certain immune cells and target genes indicates a need for larger cohorts to confirm these findings and validate the relationship between target molecules and immune responses experimentally. Future prospective studies are essential to translate these findings into clinical practice and to enhance our understanding of the molecular and immunological mechanisms underlying IPF.

## 5 Conclusion

In summary, this study identified PODNL1 and PIGA as potential biomarkers for the diagnosis of IPF and explored their possible roles in its pathogenesis. These findings contribute to a deeper understanding of the mechanisms underlying IPF and offer promising avenues for developing novel diagnostic and therapeutic strategies.

## Data Availability

The datasets presented in this study can be found in online repositories. The names of the repository/repositories and accession number(s) can be found in the article/Supplementary Material.
