# Supplementary material for: Machine learning potential predictor of idiopathic pulmonary fibrosis
Source: Front Genet. 2025 Jan 22;15:1464471. doi: 10.3389/fgene.2024.1464471 (PMC11811625; doi:10.3389/fgene.2024.1464471)
Supplement: Supplementary file 1 [file DataSheet1.docx]

Supplementary Material

# Supplementary Figures and Tables

## Supplementary Figures


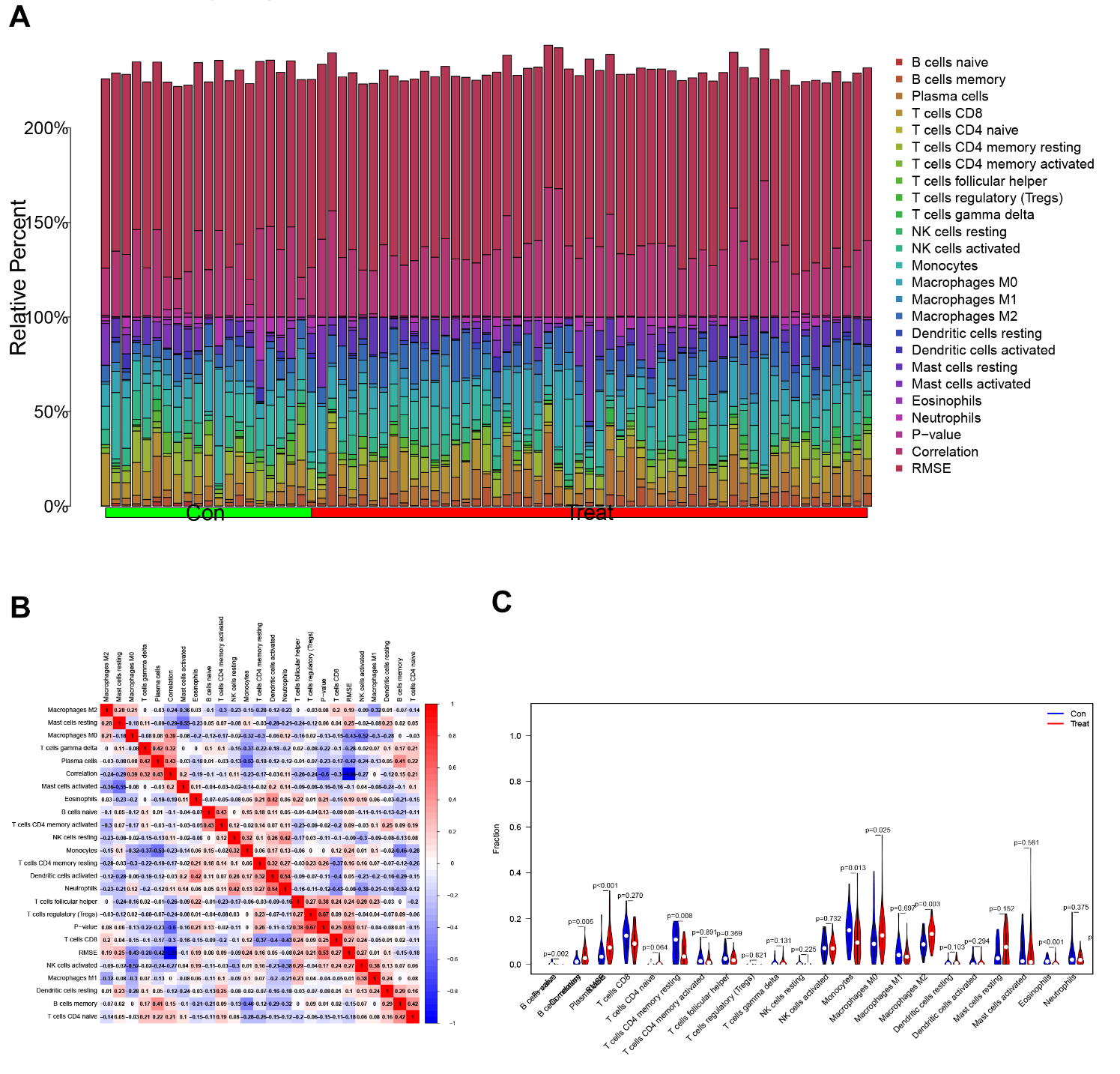


**Figure S1.** Immune infiltration: (A) Bar plot of the percentage of the 25 types of immune cells between IPF group and control group. (B) Correlation of 25 types of immune cells. (C) Violin plot of the immune cell infiltration difference demonstrated that patients with idiopathic pulmonary fibrosis.


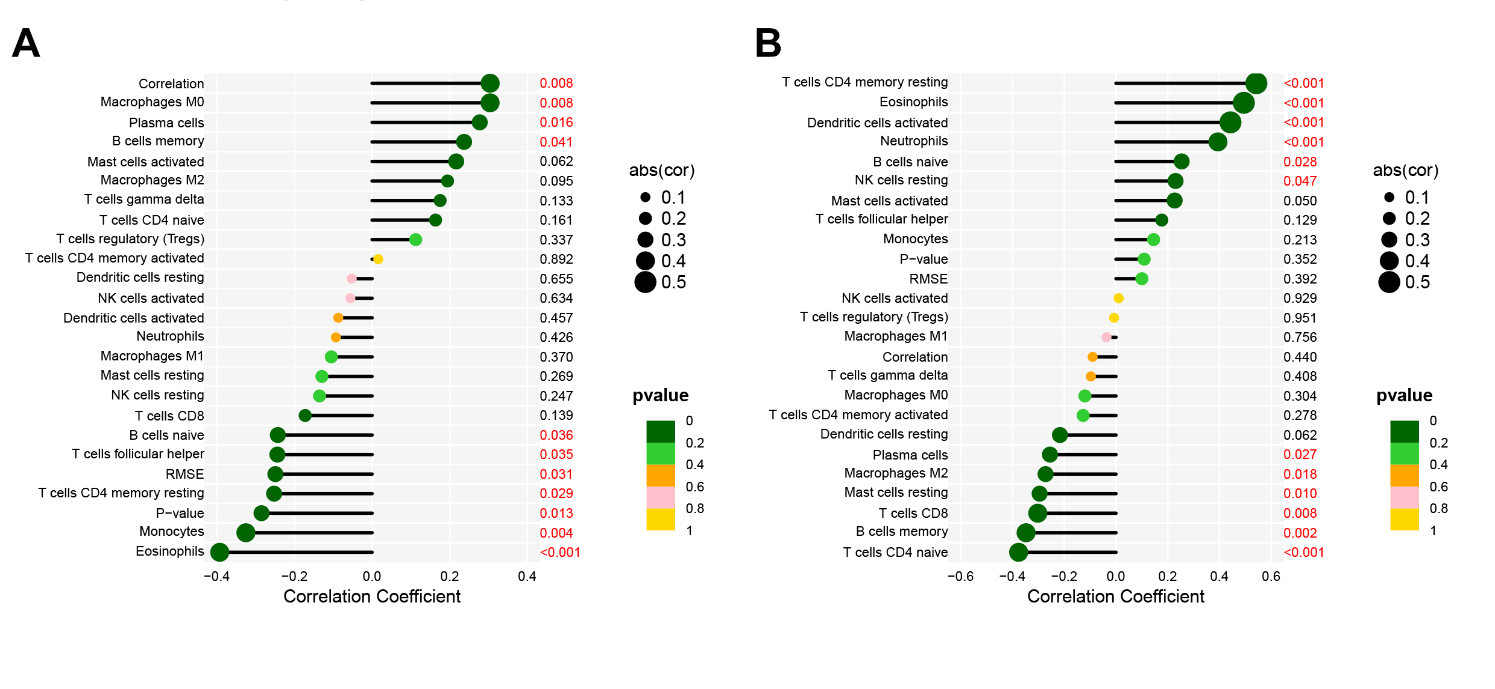


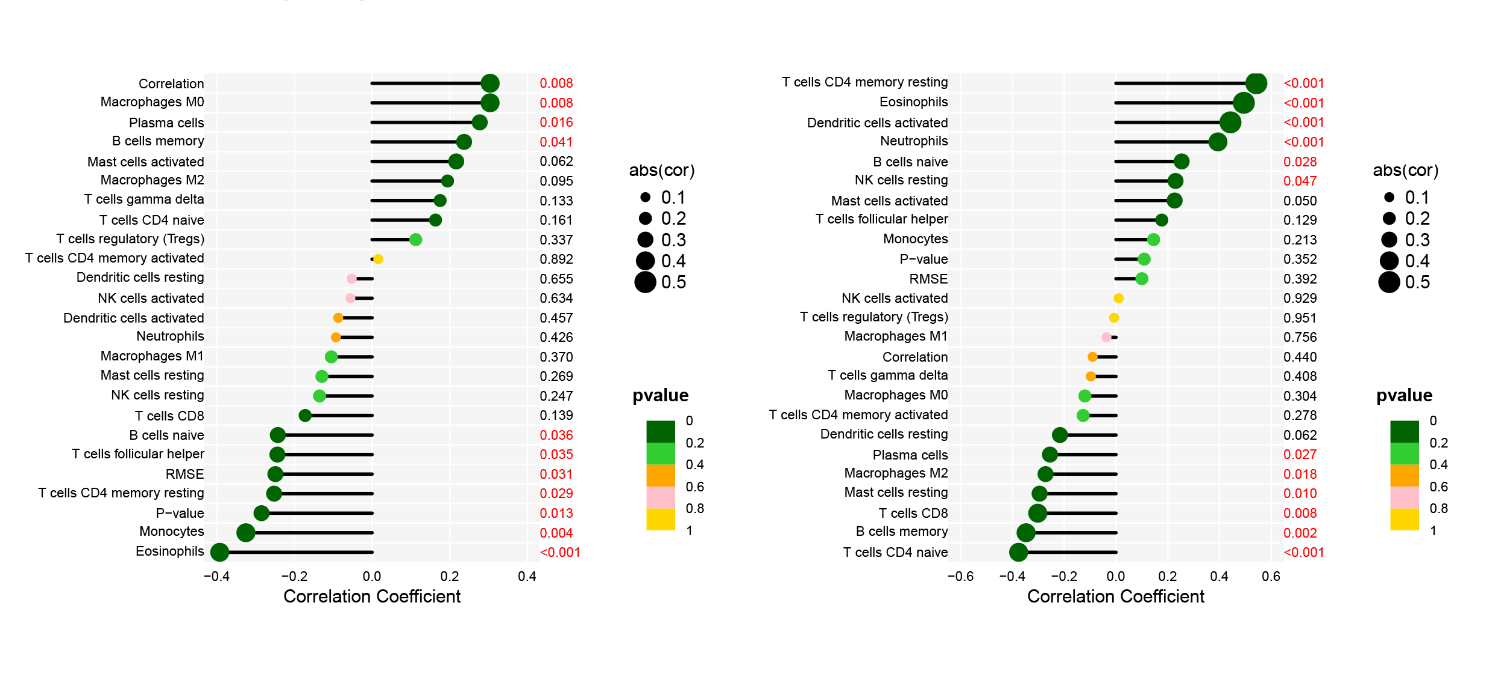


**Figure S2.** Correlation analysis between biomarkers and immune cells: (A) Correlation analysis between PODNL1 and immune cells. (B) Correlation analysis between PIGA and immune cells.


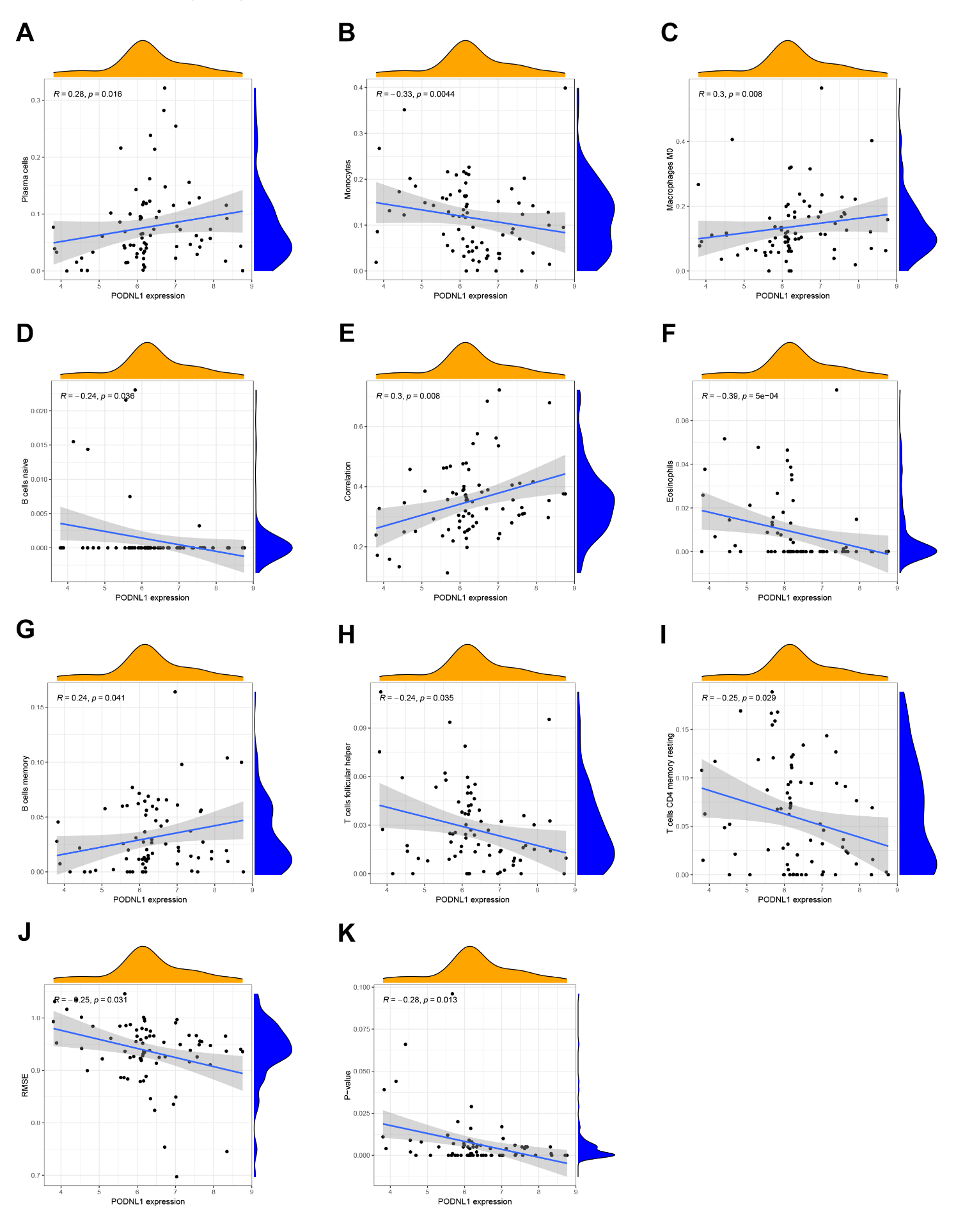


**Figure S3.** (A-K) Correlation analysis between PODNL1 and immune cells.


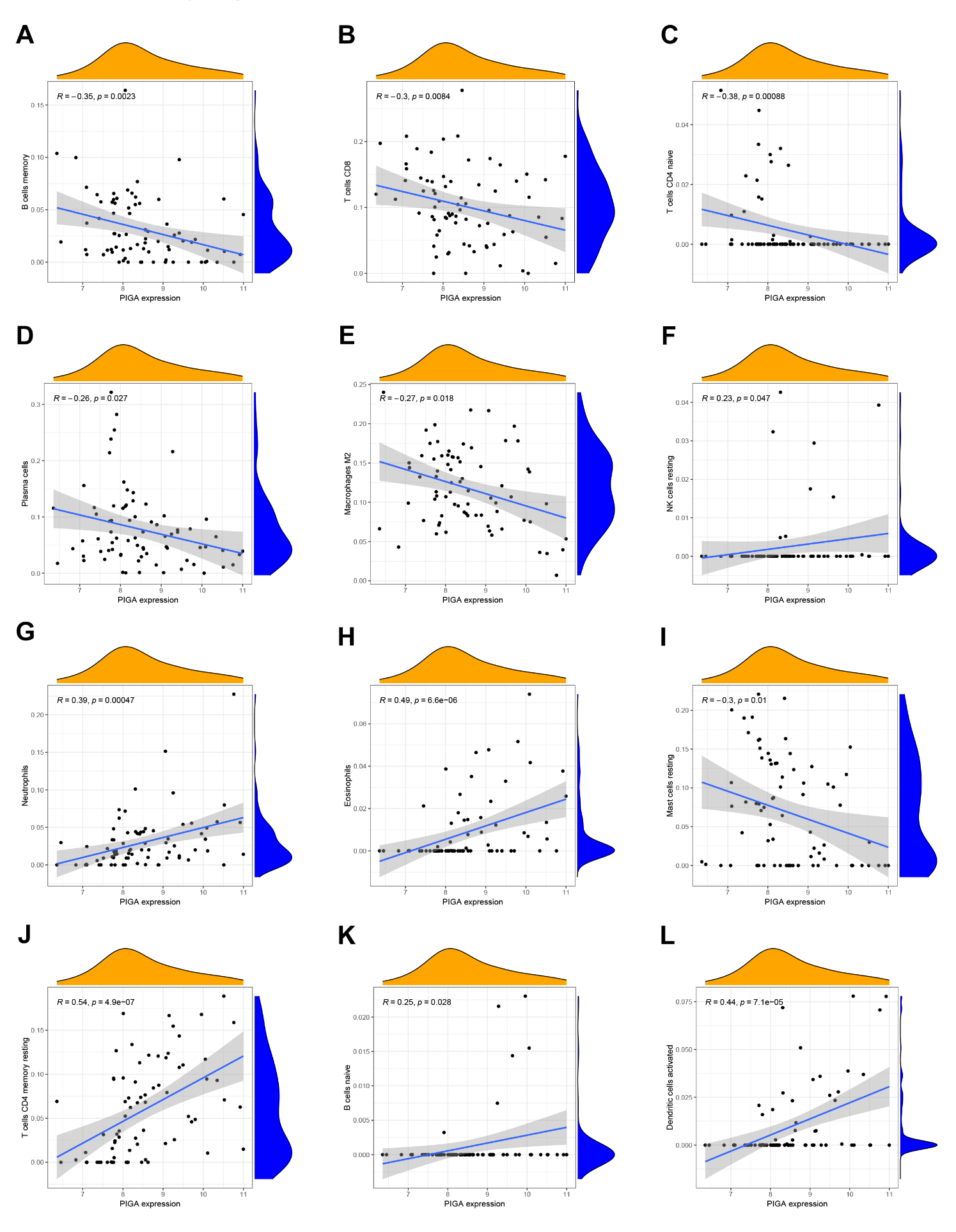


**Figure S4.** (A-L) Correlation analysis between PIGA and immune cells.
